# Effect of genetic polymorphisms of interleukin‐1 beta on the microscopic portal vein invasion and prognosis of hepatocellular carcinoma

**DOI:** 10.1002/jhbp.12009

**Published:** 2024-05-26

**Authors:** Yosuke Namba, Tsuyoshi Kobayashi, Takeshi Tadokoro, Sotaro Fukuhara, Ko Oshita, Keiso Matsubara, Naruhiko Honmyo, Shintaro Kuroda, Masahiro Ohira, Hideki Ohdan

**Affiliations:** ^1^ Department of Gastroenterological and Transplant Surgery Applied Life Sciences, Institute of Biomedical and Health Sciences Hiroshima University Hiroshima Japan

**Keywords:** circulating tumor cells, hepatocellular carcinomas, interleukin‐1 beta, microscopic portal vein invasion, single nucleotide polymorphisms

## Abstract

**Background:**

Several studies have demonstrated a relationship between genetic polymorphisms of interleukin‐1 beta (IL‐1β) and cancer development; however, their influence on cancer prognosis is unknown. In the present study, we aimed to evaluate the impact of IL‐1β single nucleotide polymorphisms on the hematogenous dissemination and prognosis of hepatocellular carcinoma.

**Methods:**

We conducted a retrospective cohort study including patients with hepatocellular carcinoma who underwent primary liver resection at our hospital between April 2015 and December 2018. The primary endpoints were overall and recurrence‐free survival. Secondary endpoints were microscopic portal vein invasion and number of circulating tumor cells.

**Results:**

A total of 148 patients were included, 32 with rs16944 A/A genotype. A/A genotype was associated with microscopic portal vein invasion and number of circulating tumor cells (*p* = .03 and .04). In multivariate analysis, A/A genotype, alpha‐fetoprotein level, and number of circulating tumor cells were associated with microscopic portal vein invasion (*p* = .01, .01, and <.01). A/A genotype, Child‐Pugh B, and intraoperative blood loss were independent predictive factors for overall survival (*p* = .02, <.01, and <.01).

**Conclusions:**

Our results indicate that the IL‐1β rs16944 A/A genotype is involved in number of circulating tumor cells, microscopic portal vein invasion, and prognosis in HCC.

## Graphical abstract

Interleukin‐1 beta gene polymorphisms affect interleukin‐1 beta production in the portal vein. Namba and colleagues found that the rs16944 A/A genotype was associated with microscopic portal vein invasion and the number of circulating tumor cells, and was an independent predictive factor for overall and recurrence‐free survival in hepatocellular carcinoma.

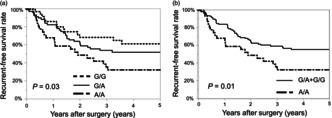

## 1 INTRODUCTION

Hepatocellular carcinoma (HCC) is one of the most common malignancies occurring worldwide and the third leading cause of cancer‐related death worldwide.^1^ Although hepatectomy is expected to be curative for HCC, the prognosis remains poor due to the high incidence of recurrence and metastasis.^2^ Therefore, identifying poor prognostic factors in HCC and finding new therapeutic targets is of paramount importance.

The inflammatory microenvironment has been shown to be closely associated with hematogenous dissemination, and various factors have been discovered by which tumor and inflammation interfere with each other.^3^, ^4^ Interleukin‐1 beta (IL‐1β) is one such example, and several reports have shown an association between IL‐1β and malignancy.^5^, ^6^ IL‐1β is known to participate in both systemic and local inflammatory processes and to mediate several immune responses.^7^, ^8^ Recently, IL‐1β has been reported to mediate HCC progression by promoting epithelial–mesenchymal transition (EMT) in cancer cells.^9^ It has also been shown that IL‐1β is involved in the immune escape mechanism of the programmed cell death‐ligand 1 (PD‐L1)/programmed cell death protein 1 (PD‐1) pathway,^10^, ^11^, ^12^ further suggesting the importance of IL‐1β.

Additionally, single‐nucleotide polymorphism (SNP) in the promoter region within the IL‐1β gene may be involved in the expression level of IL‐1β and has been reported to be associated with various diseases including malignant tumors.^13^, ^14^ Although there are also several reports associated with HCC,^15^, ^16^ these reports are on the development of malignancy, and very few reports are on prognosis. In our investigation, there was only one report of an association between IL‐1β SNP and prognosis of HCC, which was only for hepatitis C virus patients.^17^ Demonstrating an association between IL‐1β SNPs and HCC prognosis may lead to the elucidation of new therapeutic targets.

In this study, we aimed to report the impact of IL‐1β SNP on the prognosis of HCC. We further evaluated the association of IL‐1β SNP with microvascular invasion and circulating tumor cells (CTCs) in peripheral blood to examine the impact of IL‐1β on hematogenous dissemination.

## 2 METHODS

### 2.1 Patient recruitment and study protocol

**FIGURE 1:**
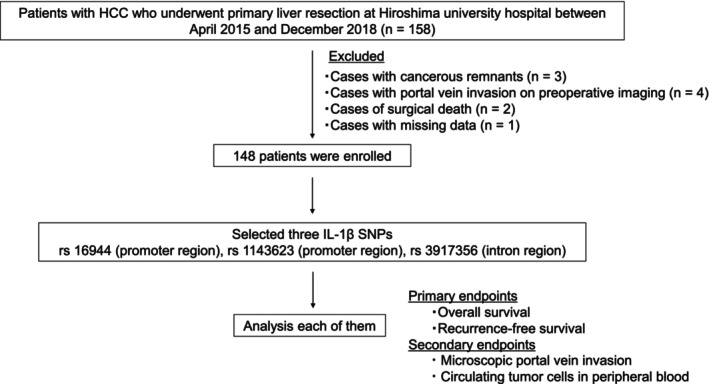
Flow chart of this study.

We conducted a retrospective cohort study including patients with HCC who underwent primary liver resection at our hospital between April 2015 and December 2018. We used the database of our hospital to extract baseline clinicopathological findings. Tumor recurrence and metastasis were determined using computed tomography or magnetic resonance imaging. The pathologist identified microscopic portal vein invasion (mPVI) through the analysis of surgical specimens. The study excluded cases that exhibited portal vein invasion on preoperative imaging, contained cancerous remnants, experienced mortality during surgery, or had incomplete data. The primary endpoints were overall survival (OS) and recurrence‐free survival (RFS). Secondary endpoints were the presence or absence of mPVI and CTCs in peripheral blood (Figure 1).

### 2.2 Data collection

We assessed patient demographics, clinical information, perioperative factors and long‐term outcomes for each case. Patient demographics included age, sex, body mass index, history of diabetes, and Child‐Pugh classification. Clinical factors included hepatitis background, preoperative blood test results, indocyanine green retention rate at 15 min, TNM stage, tumor size, the number of tumors, CTCs in peripheral blood, and the presence or absence of mPVI. TNM stage was classified using the Union for International Cancer Control TNM classification of malignant tumors, eighth edition. Long‐term outcomes evaluated the OS, RFS, and recurrence rate. Early recurrence was defined as recurrence within 1 year after surgery.

Informed consent was obtained from the recruited patients, and the Institutional Review Board of each institution provided approval for this study (E‐1639). This study was performed according to the principles of the Declaration of Helsinki and reported in line with the STROBE guideline.

### 2.3 Single nucleotide polymorphism selection and genotyping

Based on a database search (http://ncbi.nlm.nih.gov/SNP), we selected three IL‐1β SNPs (rs16944 G>A in the promoter region, rs1143623 C>G in the promoter region, and rs3917356 C>T in the intron) that have been reported to be associated with malignancy.^14^, ^18^, ^19^ Genomic deoxyribonucleic acid (DNA) was extracted from peripheral blood mononuclear cells (PBMCs) or whole blood using a QIAcube (Qiagen, Hilden, Germany). Genotyping of all SNPs was performed using TaqMan SNP genotyping assays (Thermo Fisher Scientific, MA, USA). Figure S1 shows an example of the results.

### 2.4 Identification and analysis of CTCs

We previously reported a method for measuring CTC using GPC3, which was expressed in 90% of HCC. This study used the same method to measure CTCs.^20^ CTCs were detected by immuno‐magnetic positive enrichment coupled with flow cytometry using peripheral blood immediately before surgery. CTCs were stained with allophycocyanin (APC)‐conjugated mouse anti‐human glypican‐3 (GPC3) monoclonal antibodies (clone #307801; R&D Systems Inc., USA), and sorting GPC3‐positive cells by immuno‐magnetic positive enrichment. They were stained with a phycoerythrin‐cyanine 7 (PE‐Cy7)‐conjugated mouse anti‐human CD45 antibodies (clone HI30; Becton Dickinson), fluorescein isothiocyanate (FITC)‐conjugated mouse anti‐human CD235a antibodies (clone GA‐R2 (HIR2); Becton Dickinson), and 7‐Aminoactinomycin D (7‐AAD, Becton Dickinson). In this study, we considered the GPC3 + CD45‐CD235a‐7‐AAD‐events detected by flow cytometry as CTCs. The cutoff value for CTCs in peripheral blood was set at 4 based on mPVI.

### 2.5 Measurement of serum IL‐1β production

Serum IL‐1β production was measured in a subgroup analysis. Serum samples of preoperative peripheral blood, portal vein, and hepatic venous blood were collected before liver resection for patients who underwent anatomical liver resection; thirty patients who underwent initial anatomical liver resection for HCC from April 2021 to April 2022 were included. Blood samples were centrifuged for 25 min at 1800 *g*, and the serum of each sample stored at −80°C. Serum IL‐1β levels were measured with corresponding human ELISA kits (Abcam, Shanghai, China), strictly following the manufacturer's instructions.

### 2.6 Cell culture

To evaluate the effect of IL‐1β on HCC cells, HepG2 was used as the HCC cell line. The HepG2 cell line (Cell Line Services, Heidelberg, Germany) was cultured at 37°C under 5% CO_2_ in Dulbecco's modified Eagle medium (DMEM: Seromed, Berlin, Germany) supplemented with 10% heat‐inactivated fetal calf serum (FCS), 100 IU/mL penicillin, and 100 μg/mL streptomycin (Gibco, Karlsruhe, Germany). The culture media were replaced each third day, and the cells were not used beyond passage 10 to maintain equal conditions between the experiments.

### 2.7 Stimulation protocol

To confirm the responsibility of the IL‐1β for the EMT or expression of PD‐L1 in HCC cells, cytokine treatment was performed. HepG2 cells were pretreated with recombinant IL‐1β (50 ng/mL, 200‐01B, Peprotech, UK) for 48 h. The expression of vimentin and PD‐L1 were detected using real‐time quantitative reverse transcription–polymerase chain reaction (qRT‐PCR).

### 2.8 qRT‐PCR

Total ribonucleic acid (RNA) was prepared from the HepG2 cells using RNeasy Mini kits (Qiagen, Limburg, The Netherlands) and reverse transcribed using the ReverTra Ace qPCR RT Kit (Toyobo Life Science, Tokyo, Japan) according to the manufacturer's instructions. The resulting cDNA was amplified with Rotor‐Gene 3000 and Rotor‐Gene SYBR Green PCR Kit (Qiagen) and gene‐specific primers for vimentin (NM 003380) and CD274 (NM 014143). β‐actin (NM 001101) was used as an internal control. Details of each primer are shown in Table S1. Data were analyzed using the ΔCt method for relative quantification.

### 2.9 Statistical analysis

Data were analyzed using JMP software version 16 (SAS Institute, Inc., Cary, NC, USA). Descriptive statistics for categorical and continuous variables are reported as absolute numbers and median (range), respectively. Categorical variables were analyzed using Fisher's exact test, and continuous variables using student's *t*‐test for normal distribution and Wilcoxon test for non‐normal distribution. Analysis of variance (ANOVA) was used to analyze continuous variables among the three groups. OS and RFS rates were calculated using the Kaplan–Meier method and compared using the log‐rank test. The multivariate analyses for the variables independently related to the OS and the RFS using the Cox proportional hazard model were carried out. The multiple logistic regression model was used to identify the independent predictors of mPVI. All variables were included in the multivariate models and the backward elimination method with removal criterion *p* = .05 was used to select covariates. The receiver operating characteristic (ROC) curve was used to determine the cutoff value for each continuous variable. Statistical significance was set at *p* < .05. The control subjects were checked for Hardy–Weinberg equilibrium (HWE) using a chi‐square goodness‐of‐fit test.

## 3 RESULTS

### 3.1 Relationship between patient characteristics and IL‐1β genetic polymorphisms

In total, 148 patients were included in this study. All genotype distributions were consistent with HWE (*p* > .05, Table S2). The relationship between the genotypic distribution of IL‐1β rs16944 and the baseline clinical characteristics is shown in Table 1. There were no significant differences in the background factors. In terms of tumor factors, tumor size and number were not significantly different. Number of CTCs and mPVI was significantly more common in the mutant genotype, A/A (*p* = .04 and *p* = .03, respectively). Regarding the recurrence pattern, there was no significant difference in early recurrence or extrahepatic recurrence; however intrahepatic recurrence was more common in the A/A genotype (*p* = .04). In rs1143623 and rs3917356, no significant differences were observed for all of the factors (Tables S3 and S4).

**TABLE 1:** The relationship between the genotypic distribution of IL‐1β rs16944 and the baseline clinical characteristics.

	A/A *N* = 32 (21.7%)	G/A *N* = 76 (51.3%)	G/G *N* = 40 (27.0%)	*p*‐value
Age (years), mean (95% CI)	71.0 (68.4–73.6)	72.4 (70.7–74.2)	71.2 (69.2–73.2)	.55
Male, *n* (%)	25 (78.1)	59 (77.6)	30 (75.0)	.93
BMI (kg/m^2^), mean (95% CI)	24.2 (23.2–25.3)	23.4 (22.7–24.2)	23.4 (22.6–24.3)	.41
HBV/HCV, *n* (%)	18 (56.2)	43 (56.5)	26 (65.0)	.43
Child‐Pugh B, *n* (%)	5 (15.7)	6 (7.9)	2 (5.0)	.15
ICG‐R15 (%), mean (95% CI)	16.9 (12.5–21.2)	17.2 (14.3–20.2)	17.1 (13.7–20.6)	.99
AFP (ng/mL), mean (95% CI)	183 (−6376 to 6743)	3378 (−983 to 7740)	1565 (−3628 to 6759)	.70
DCP (mAU/mL), mean (95% CI)	5625 (−12 856–24 107)	12 694 (406–24 983)	3953 (−10 679 to 18 587)	.63
Multiple tumors, *n* (%)	12 (37.5)	19 (25.0)	12 (30.0)	.52
Tumor size (mm), mean (95% CI)	36.9 (27.0–46.7)	38.0 (31.5–44.5)	35.3 (27.5–43.1)	.87
mPVI, *n* (%)	13 (40.6)	16 (21.0)	7 (17.5)	.03
mHVI, *n* (%)	2 (6.2)	7 (9.2)	3 (7.6)	.86
Number of CTCs ≥4, *n* (%)	18 (56.2)	32 (42.1)	11 (27.5)	.04
Early recurrence, *n* (%)	7 (21.8)	11 (14.4)	6 (15.0)	.76
Intrahepatic recurrence, *n* (%)	19 (59.3)	27 (35.5)	13 (32.5)	.04
Extrahepatic recurrence, *n* (%)	6 (18.7)	11 (14.4)	5 (12.5)	.84

Abbreviations: AFP, alpha fetoprotein; BMI, body mass index; CI, confidence interval; CTCs, circulating tumor cells; DCP, des‐γ‐carboxy prothrombin; HBV, hepatitis B virus; HCV, hepatitis C virus; ICG‐R15, indocyanine green retention rate at 15 min; mPVI, microscopic portal vein invasion mHVI, microscopic hepatic vein invasion.

### 3.2 Correlation between IL‐1β rs16944 a allele and incidence of mPVI

The relationship between the incidence of mPVI and clinical factors, including IL‐1β gene polymorphism is shown in Table 2. Univariate analysis revealed that rs16944 A/A genotype (*p* < .01), alpha‐fetoprotein (AFP) level (*p* < .01), des‐gamma‐carboxyprothrombin (DCP) level (*p* = .03), tumor size (*p* < .01), and number of CTCs were associated with mPVI. Multivariate analysis identified rs16944 A/A genotype (odds ratio [OR]: 3.38; 95% confidence interval [CI]: 1.60–7.17; *p* = .01), AFP level (odds ratio [OR]: 3.10; 95% confidence interval [CI]: 1.21–7.93; *p* = .01), and number of CTCs (odds ratio [OR]: 7.50; 95% confidence interval [CI]: 2.70–20.8; *p* < .01) as independent risk factors for mPVI.

**TABLE 2:** Risk factors for microscopic portal vein invasion.

	Univariate analysis		Multivariate analysis	
	OR (95% CI)	*p*‐value	OR (95% CI)	*p*‐value
Age >70 years	0.60 (0.30–1.17)	.13		
Male	1.21 (0.53–2.73)	.64		
HBV/HCV	0.83 (0.27–2.59)	.75		
Child‐Pugh B	2.11 (0.80–6.66)	.08		
ICG‐R15 >15%	0.81 (0.40–1.60)	.54		
AFP >20 ng/mL	4.61 (2.23–9.53)	<.01	3.10 (1.21–7.93)	.01
DCP >40 mAU/mL	2.88 (1.04–4.04)	.03		
Multiple tumors	1.80 (0.89–3.62)	.09		
Tumor size >50 mm	4.34 (2.14–8.78)	<.01		
Number of CTCs ≥4	8.44 (3.18–22.3)	<.01	7.50 (2.70–20.8)	<.01
rs16944 A/A genotype	3.38 (1.60–7.17)	<.01	3.36 (1.23–9.18)	.01

Abbreviations: AFP, alpha fetoprotein; CI, confidence interval; CTCs, circulating tumor cells; DCP, des‐γ‐carboxy prothrombin; HBV, hepatitis B virus; HCV, hepatitis C virus; ICG‐R15, indocyanine green retention rate at 15 min; mPVI, microscopic portal vein invasion; OR, odds ratio.

### 3.3 IL‐1beta genetic polymorphisms and overall survival

**FIGURE 2:**
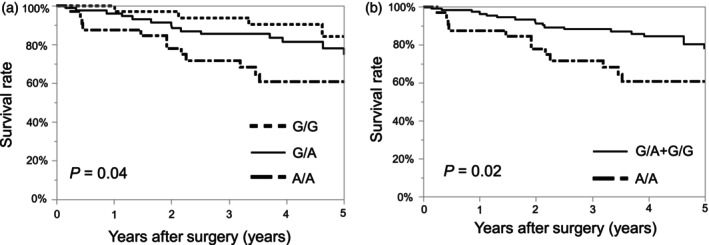
Overall survival curve according to the genotypic distribution of IL‐1β rs16944. (a) Kaplan–Meier survival curve analysis showed significantly worse overall survival in the A/A genotype than in the other groups. (b) In the recessive model, the A/A genotype was a significant risk factor for lower overall survival.

**FIGURE 3:**
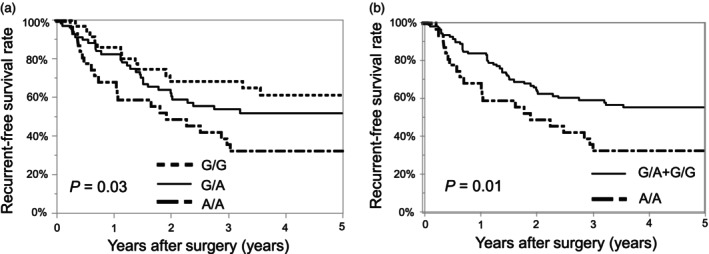
Recurrence‐free survival curve according to the genotypic distribution of IL‐1β rs16944. (a) The recurrence‐free survival of A/A genotype was significantly lower than that of the other genotypes. (b) The recessive model also showed significantly lower recurrence‐free survival for the A/A genotype.

The relationship between the genotypic distribution of IL‐1β rs16944 and OS is shown in Figure 2. Kaplan–Meier survival curve analysis showed that the OS of A/A genotype was significantly lower than that of the other groups (*p* = .04, Figure 2a). In the recessive model, the A/A genotype was a significant risk factor for lower OS (*p* = .02, Figure 2b). The relationship between genetic polymorphisms IL‐1β rs16944 and RFS is shown in Figure 3. The RFS of A/A genotype was significantly lower than that of the other genotypes (*p* = .03, Figure 3a), and the recessive model also showed significantly lower RFS for the A/A genotype (*p* = .01, Figure 3b). For rs1143623 and rs3917356, OS and RFS were not significantly different (Figures S2 and S3).

### 3.4 Risk factors for the overall and recurrence‐free survival

The risk factors of poor OS are shown in Table 3. Univariate analysis revealed that that rs16944 A/A genotype (*p* < .01), Child‐Pugh B (*p* < .01), albumin level (*p* < .01), DCP level (*p* = .02), multiple tumors (*p* < .01), tumor size (*p* < .01), operation time (*p* = .03), blood loss (*p* < .01), number of CTCs (*p* < .01), and mPVI (*p* < .01) were predictive factors for OS. In the multivariate analysis, rs16944 A/A genotype (OR: 2.48; 95% CI: 1.15–5.35; *p* = .02), Child‐Pugh B (OR: 5.08; 95% CI: 2.11–12.2; *p* < .01), and blood loss (OR: 4.81; 95% CI: 2.11–10.9; *p* < .01) were independent predictive factors for the OS. With respect to risk factors for poor RFS, rs16944 A/A genotype (*p* = .03), Child‐Pugh B (*p* < .01), AFP level (*p* < .01), DCP level (*p* < .01), multiple tumors (*p* < .01), tumor size (*p* < .01), operation time (*p* = .03), blood loss (*p* < .01), number of CTCs (*p* < .01), and mPVI (*p* < .01) were predictive factors in the univariate analysis. In multivariate analysis, Child‐Pugh B (OR: 2.53; 95% CI: 1.17–5.48; *p* < .01), multiple tumors (OR: 2.80; 95% CI: 1.62–4.82; *p* < .01), tumor size (OR: 2.06; 95% CI: 1.15–3.68; *p* = .01), and mPVI (OR: 2.58; 95% CI: 1.51–4.40; *p* < .01) were independent predictive factors of poor RFS (Table 4).

**TABLE 3:** Risk factors for overall survival.

	Univariate analysis		Multivariate analysis	
	OR (95% CI)	*p*‐value	OR (95% CI)	*p*‐value
Age >70 years	1.32 (0.68–2.56)	.40		
Male	1.24 (0.66–2.50)	.45		
HBV/HCV	0.54 (0.28–1.05)	.07		
Child‐Pugh B	6.25 (2.66–14.6)	<.01	5.08 (2.11–12.2)	<.01
ICG‐R15 >15%	1.32 (0.69–2.55)	.39		
AFP >20 ng/mL	1.93 (0.99–3.76)	.05		
DCP >40 mAU/mL	2.35 (1.10–5.01)	.02		
Multiple tumors	2.67 (1.38–5.18)	<.01		
Tumor size >30 mm	3.92 (2.02–7.60)	<.01		
Operation time >300 min	2.19 (1.00–4.78)	.04		
Blood loss >500 mL	4.36 (2.00–9.48)	<.01	4.81 (2.11–10.9)	<.01
Number of CTCs ≥4	2.83 (1.33–6.02)	<.01		
mPVI	3.94 (2.03–7.63)	<.01		
rs16944 A/A genotype	2.15 (1.08–4.28)	<.01	2.48 (1.15–5.35)	.02

Abbreviations: AFP, alpha fetoprotein; CI, confidence interval; CTCs, circulating tumor cells; DCP, des‐γ‐carboxy prothrombin; HBV, hepatitis B virus; HCV, hepatitis C virus; ICG‐R15, indocyanine green retention rate at 15 min; mPVI, microscopic portal vein invasion; OR, odds ratio.

**TABLE 4:** Risk factors for recurrence‐free survival.

	Univariate analysis		Multivariate analysis	
	OR (95% CI)	*p*‐value	OR (95% CI)	*p*‐value
Age >70 years	1.13 (0.72–1.77)	.58		
Male	1.44 (0.80–2.57)	.44		
HBV/HCV	0.65 (0.41–1.02)	.06		
Child‐Pugh B	3.11 (1.48–6.55)	<.01	2.53 (1.17–5.48)	.01
ICG‐R15 >15%	1.23 (0.79–1.92)	.35		
AFP >20 ng/mL	1.79 (1.15–2.81)	<.01		
DCP >40 mAU/mL	2.37 (1.45–3.90)	<.01		
Multiple tumors	2.69 (1.68–4.29)	<.01	2.80 (1.62–4.82)	<.01
Tumor size >50 mm	2.43 (1.48–4.29)	<.01	2.06 (1.15–3.68)	.01
Operation time >300 min	1.56 (0.95–2.54)	.07		
Blood loss >500 mL	1.97 (1.22–3.16)	<.01		
Number of CTCs ≥4	2.83 (1.33–6.02)	<.01		
mPVI	2.87 (1.70–4.83)	<.01	2.58 (1.51–4.40)	<.01
rs16944 A/A genotype	1.72 (1.04–2.84)	.03		

Abbreviations: AFP, alpha fetoprotein; CI, confidence interval; CTCs, circulating tumor cells; DCP, des‐γ‐carboxy prothrombin; HBV, hepatitis B virus; HCV, hepatitis C virus; ICG‐R15, indocyanine green retention rate at 15 min; mPVI, microscopic portal vein invasion; OR, odds ratio.

### 3.5 Relationship between IL‐1beta gene polymorphisms and IL‐1β production and mRNA expression of vimentin or PD‐L1

The relationship between IL‐1β gene polymorphisms and IL‐1β production in preoperative peripheral blood, portal vein, and hepatic venous bloods before liver resection is shown in Figure S4. In peripheral and hepatic venous bloods, there was no significant difference between IL‐1β gene polymorphisms and IL‐1β production. However, in portal vein, IL‐1β production was significantly increased in the A/A genotype (Figure S4). The relationship between IL‐1β and mRNA expression of vimentin or PD‐L1 is shown in Figure S5. Vimentin and PD‐L1 mRNA expression in HepG2 was significantly increased by IL‐1β stimulation (Figure S5).

## 4 DISCUSSION

This study showed that the IL‐1β A allele was significantly associated with mPVI and CTCs in peripheral blood and was an independent risk factor for decreased survival. Although some reports have shown a relationship between IL‐1β polymorphisms and the development of malignant tumors, reports showing a relationship with prognosis are extremely rare. To the best of our knowledge, this study is the first to show that IL‐1β genetic polymorphisms are associated with mPVI and peripheral blood CTC counts, which may help elucidate the mechanism and treatment of hematogenous dissemination in HCC.

According to previous reports, IL‐1β rs16944 AA genotype was found in approximately about 15%–22% in healthy individuals and 18%–22% in patients with cancer.^13^, ^21^ Although there is variation among reports, our results were consistent with prior literature. IL‐1β rs16944 is located in the promoter region, and SNP are thought to affect IL‐1β transcription by inhibiting or allowing the fixation of transcription factors.^20^ The T allele of rs1143627, which shows linkage disequilibrium with rs16944, has been shown to have five‐fold higher binding activity to the transcription factor compared to the C allele.^22^ As a result, IL‐1β gene expression was elevated in human monocytes, and lung tissue for the T allele of rs1143627.^23^, ^24^ Similarly, in rs16944, the A allele was shown to be involved in IL‐1β expression levels,^25^ and the rs16944 gene polymorphism in the promoter region is thought to affect the expression level of IL‐1β.

There are several reports on IL‐1β genetic polymorphisms and the development of HCC, and most of these reports have shown an association with HCC, which occurs incidentally to hepatitis B virus and hepatitis C virus.^16^, ^21^, ^26^ Generally, IL‐1β is known to be part of the NOD‐like receptor family pyrin domain containing three inflammasome, which is involved in the upregulation of inflammation and liver fibrosis markers.^27^ Therefore, it has been speculated that IL‐1β genetic polymorphisms promote IL‐1β expression and the development of HCC through inflammation and liver fibrosis. However, it has recently been shown that IL‐1β may be involved in malignant tumor progression by other mechanisms. First, in pancreatic cancer and HCC, IL‐1β has been shown to be involved in PD‐L1 expression via hypoxia‐inducible factor‐1α.^9^, ^28^ It has also been reported that IL‐1β is involved in PD‐1 expression in lung cancer,^11^ suggesting that IL‐1β may be involved in the immune escape mechanism of malignant tumors. Other studies have shown that IL‐1β is associated with tumor cell migration ability and EMT.^9^, ^29^ These indicate that IL‐1β is involved in tumor promotion by immune escape and EMT as well as inflammation and liver fibrosis. The finding that the IL‐1β A/A genotype is associated with mPVI and CTC numbers in HCC verifies previous studies and emphasizes the importance of IL‐1β gene polymorphisms in HCC treatment. This study showed that IL‐1β gene polymorphisms are associated with IL‐1β production only in the portal vein, and that IL‐1β is associated with vimentin and PD‐L1 mRNA expression in HepG2. These results suggest that IL‐1β gene polymorphisms may have a significant impact on EMT and PD‐L1 expression in the tumor microenvironment, mainly in the portal vein. Furthermore, the involvement of IL‐1β gene polymorphisms in IL‐1β production only in the portal vein may explain the finding that IL‐1β gene polymorphisms are only associated with intrahepatic recurrence. This result could also explain the result that IL‐1β gene polymorphisms are associated with RFS but not with OS.

Clinical trials with canakinumab, an IL‐1β monoclonal antibody, have been conducted in lung cancer; however, the CANOPY‐1 phase III clinical trial has not shown a survival benefit.^30^ Although the efficacy of canakinumab against malignancies is still under investigation, we believe that focusing on the genetic polymorphism of IL‐1β may lead to the development of new therapeutic strategies.

The present study had several limitations. First, the study was retrospective. Second, the number of cases analyzed for IL‐1β gene polymorphisms and IL‐1β production was small. Further studies are needed to develop new therapeutic strategies for HCC.

In conclusion, our results indicate that the IL‐1β rs16944 A/A genotype is involved in mPVI, CTCs in peripheral blood, and prognosis in HCC. Our results may lead to the development of new therapeutic strategies in HCC.

## FUNDING INFORMATION

This work was supported by Japan Agency for Medical Research and Development under grant no. JP23fk0210108 to Hideki Ohdan. The funders had no role in study design, data collection and analysis, decision to publish, or preparation of the manuscript.

## CONFLICT OF INTEREST STATEMENT

The authors have no competing interests in this study.

## Supporting information


Figure S1.

Figure S2.

Figure S3.

Figure S4.

Figure S5.

Table S1.

Table S2.

Table S3.

Table S4.

## Data Availability

The data that support the findings of this study are available on request from the corresponding author. The data are not publicly available due to privacy or ethical restrictions.
