# Supplementary figures and images for: Effect of genetic polymorphisms of interleukin‐1 beta on the microscopic portal vein invasion and prognosis of hepatocellular carcinoma
Source: J Hepatobiliary Pancreat Sci. 2024 May 26;31(8):528–36. doi: 10.1002/jhbp.12009 (PMC11503458; doi:10.1002/jhbp.12009)

Figure S1. Example of TaqMan SNP genotyping.

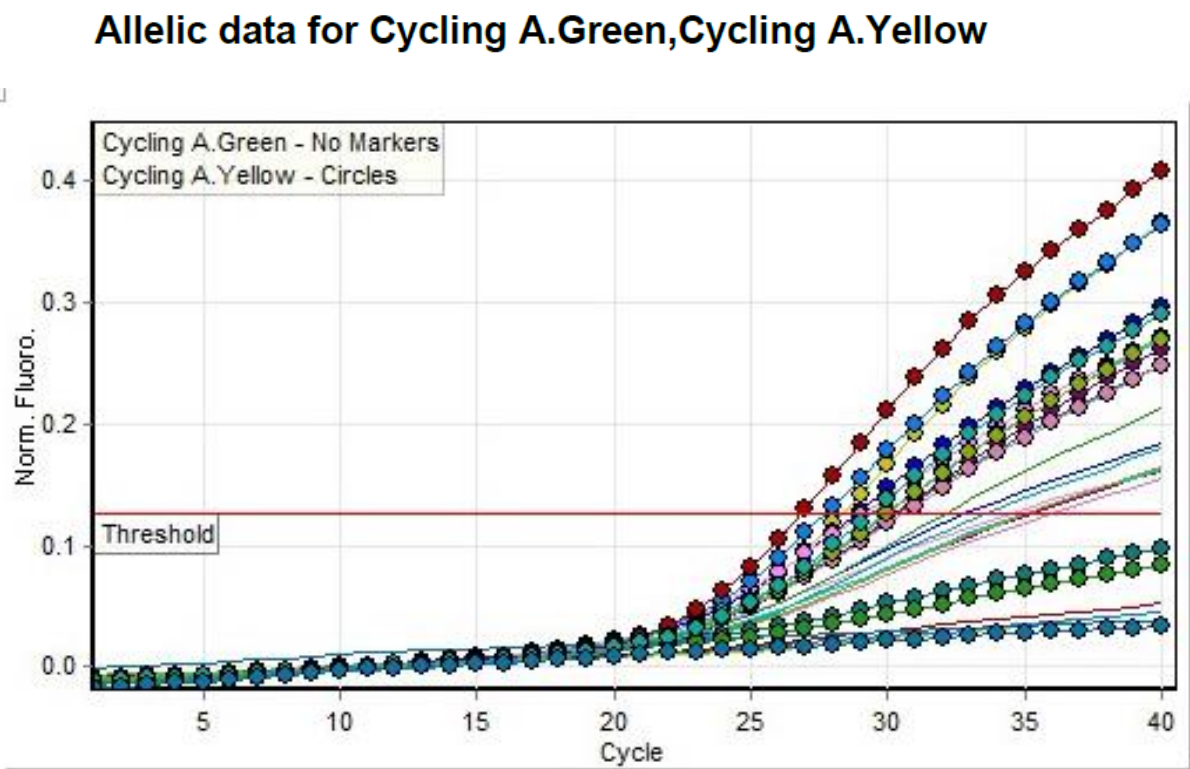

**Scatter Graph for Cycling A.Green,Cycling A.Yellow**

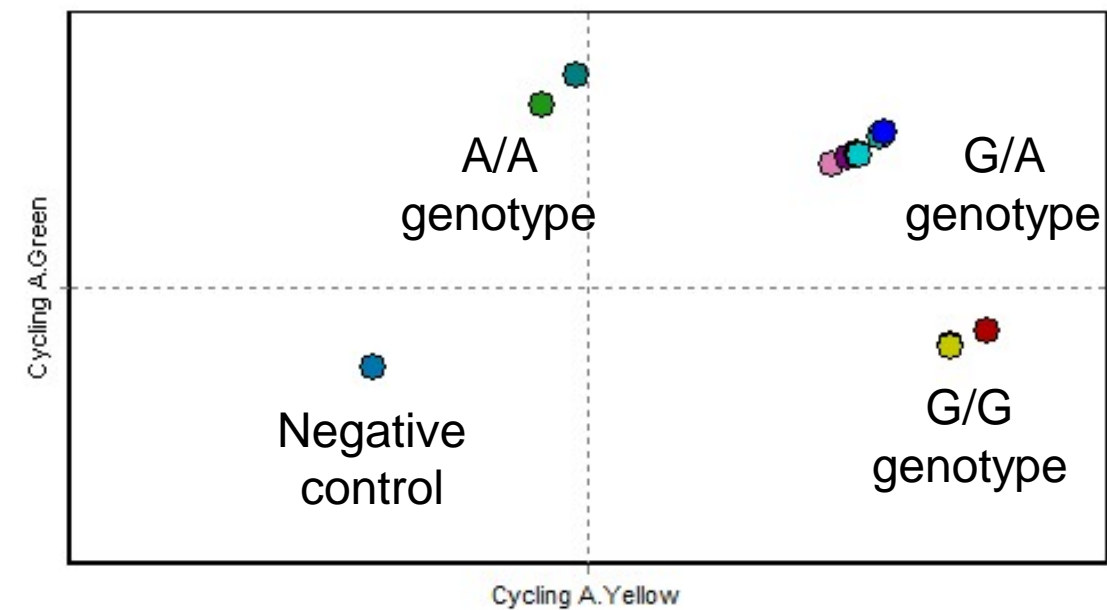

Supplement: Supplementary file 1 — Figure S1. [file JHBP-31-528-s004.pdf]

Figure S2. The relationship between the genotype of IL-1 $\beta$  rs 1143623 and survival rate.

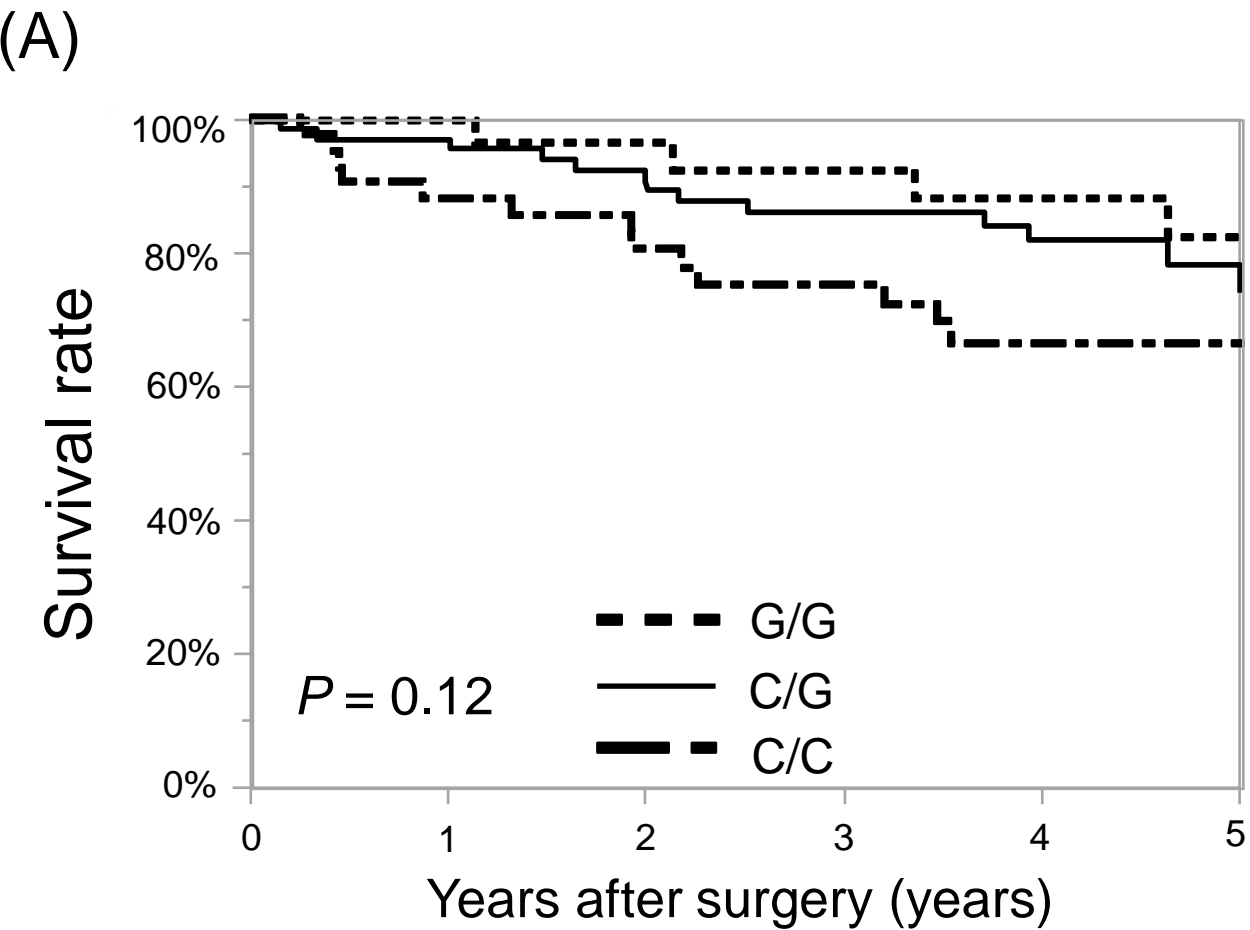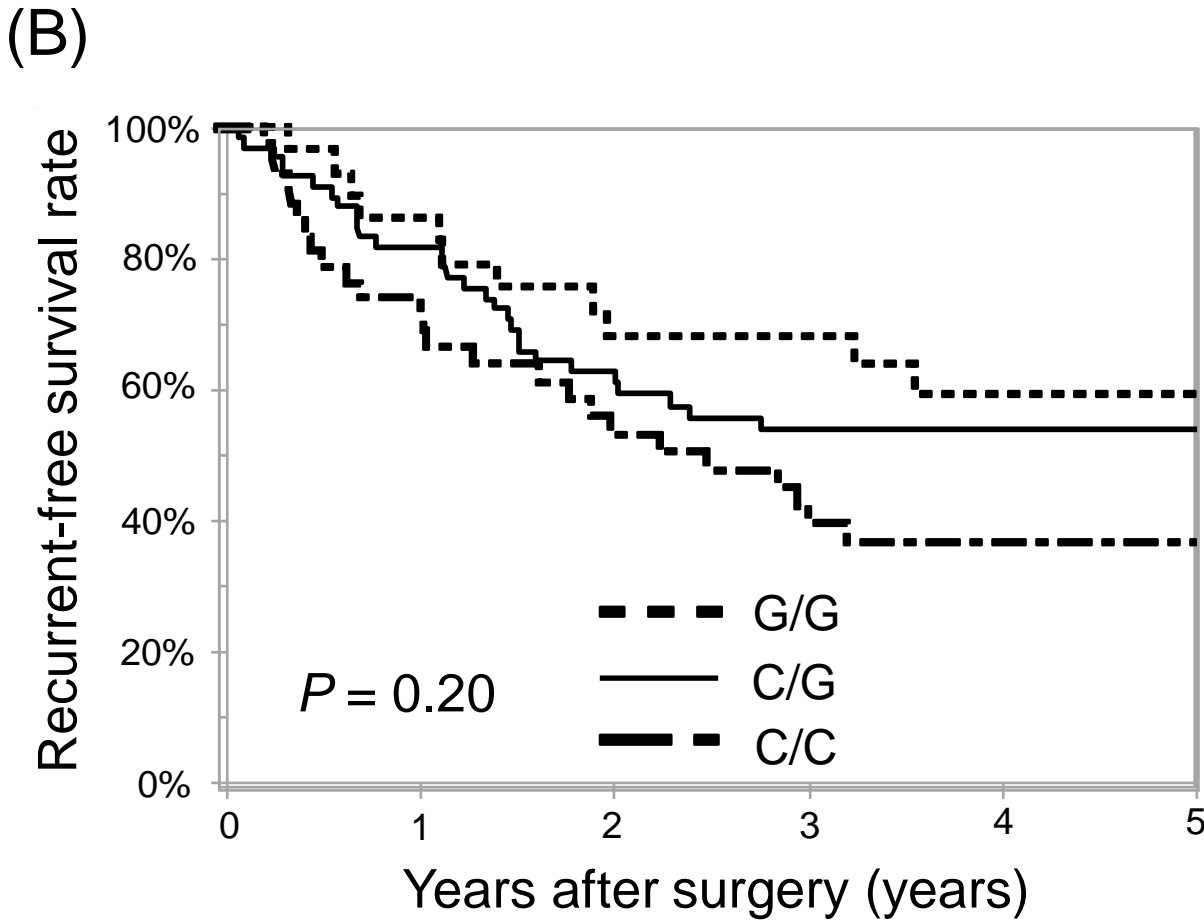

Supplement: Supplementary file 2 — Figure S2. [file JHBP-31-528-s005.pdf]

Figure S3. The relationship between the genotype of IL-1 $\beta$  rs 3917356 and survival rate.

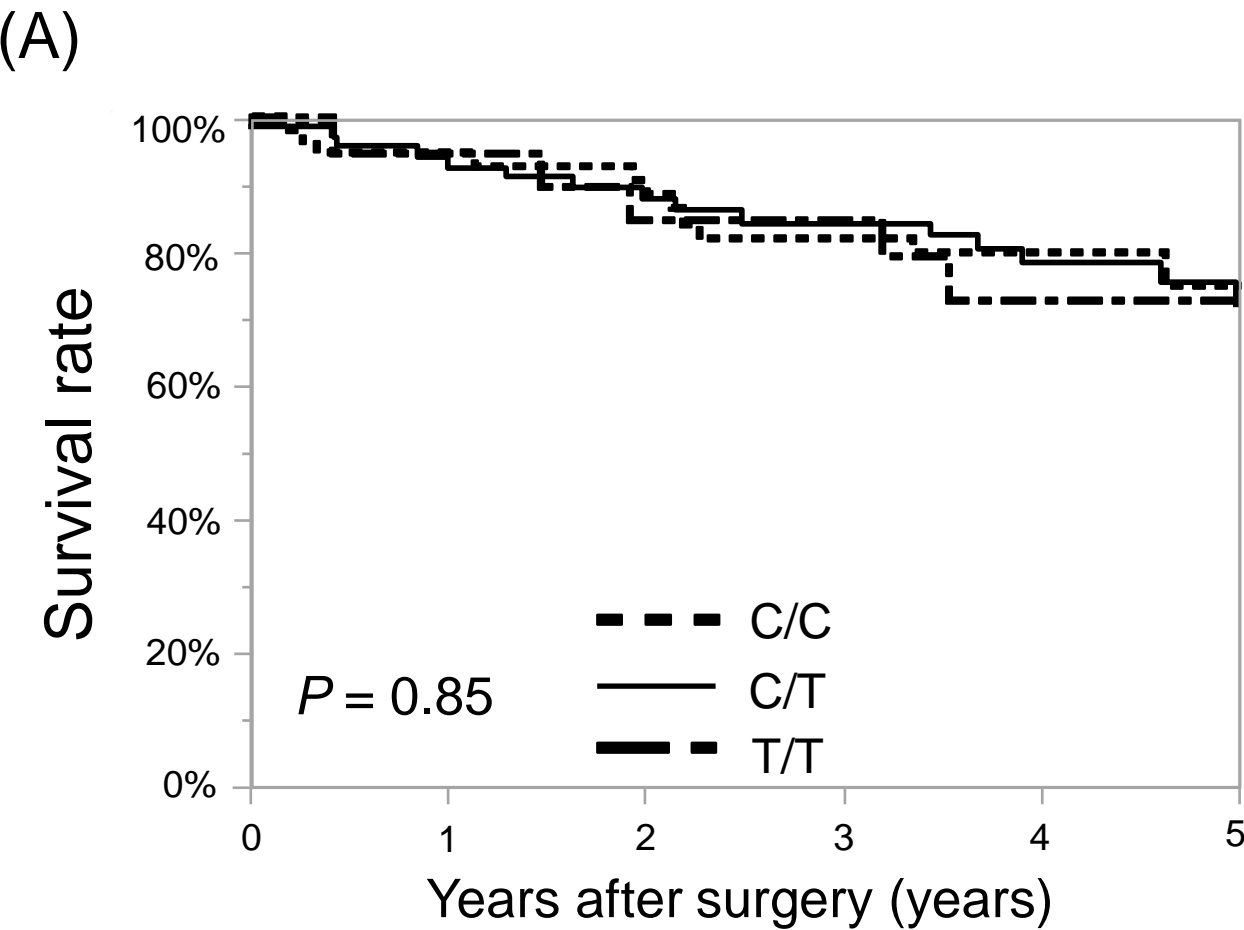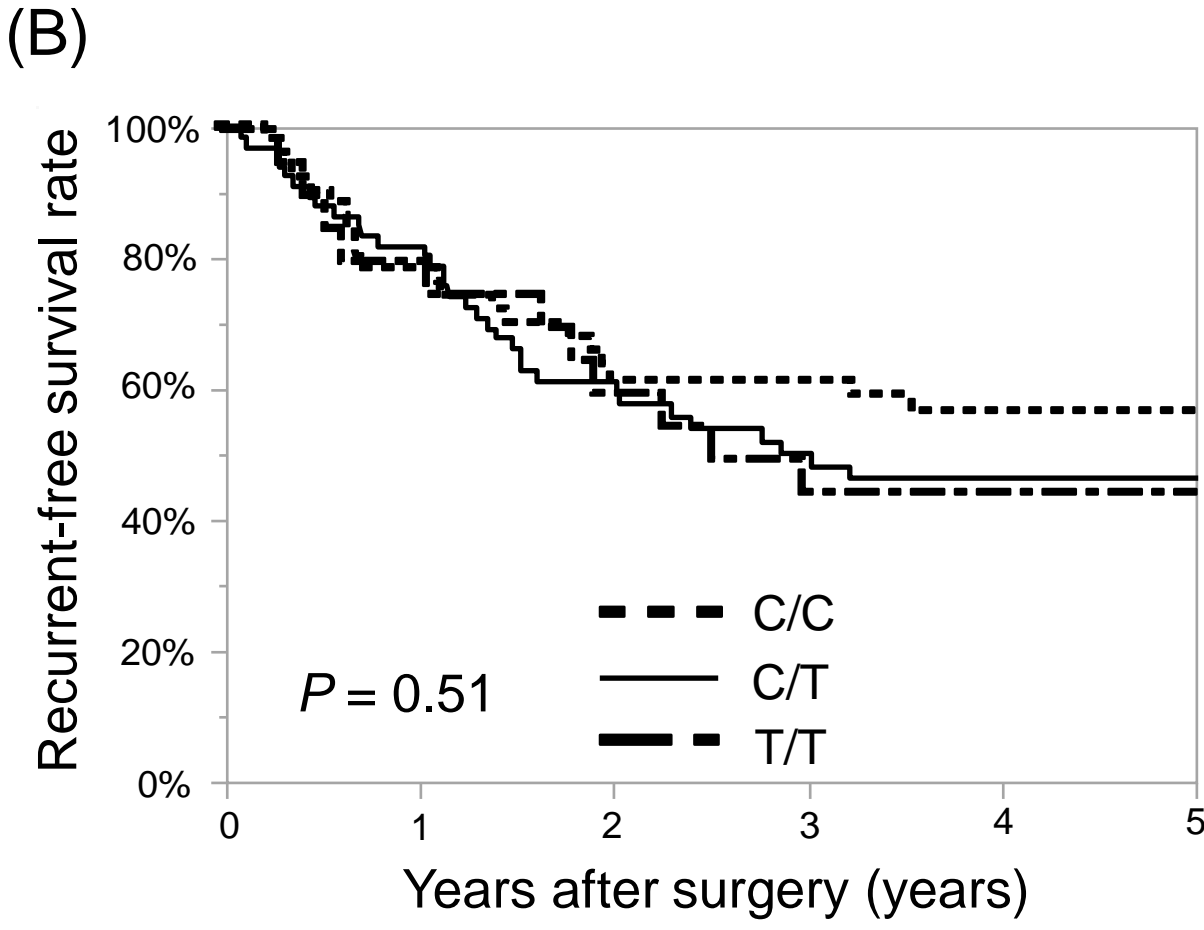

Supplement: Supplementary file 3 — Figure S3. [file JHBP-31-528-s007.pdf]

Figure S4. The relationship between IL-1 $\beta$  gene polymorphisms and IL-1 $\beta$  production.

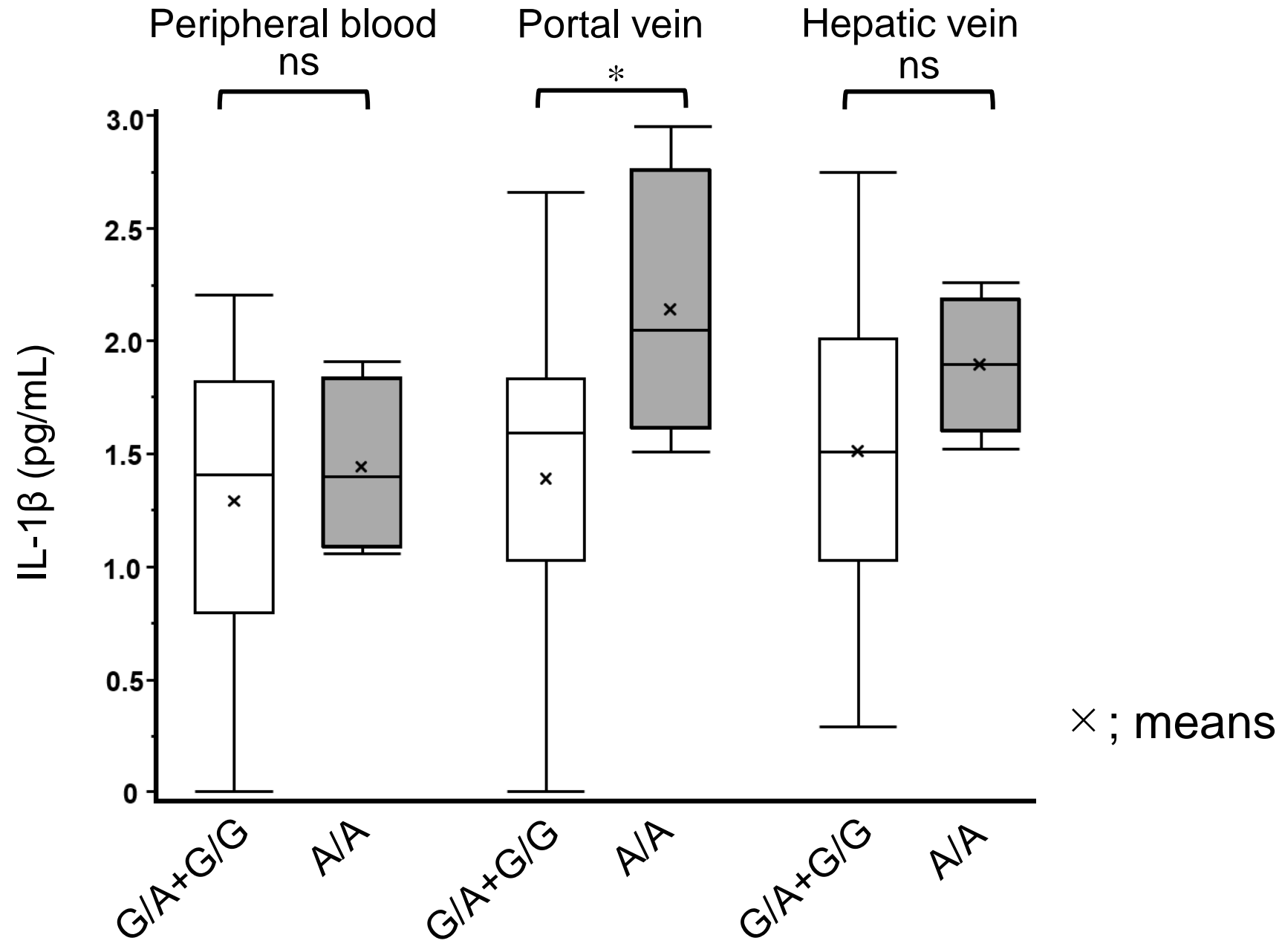

Supplement: Supplementary file 4 — Figure S4. [file JHBP-31-528-s009.pdf]

Figure S5. The relationship between IL-1 $\beta$  and Vimentin or PD-L1.

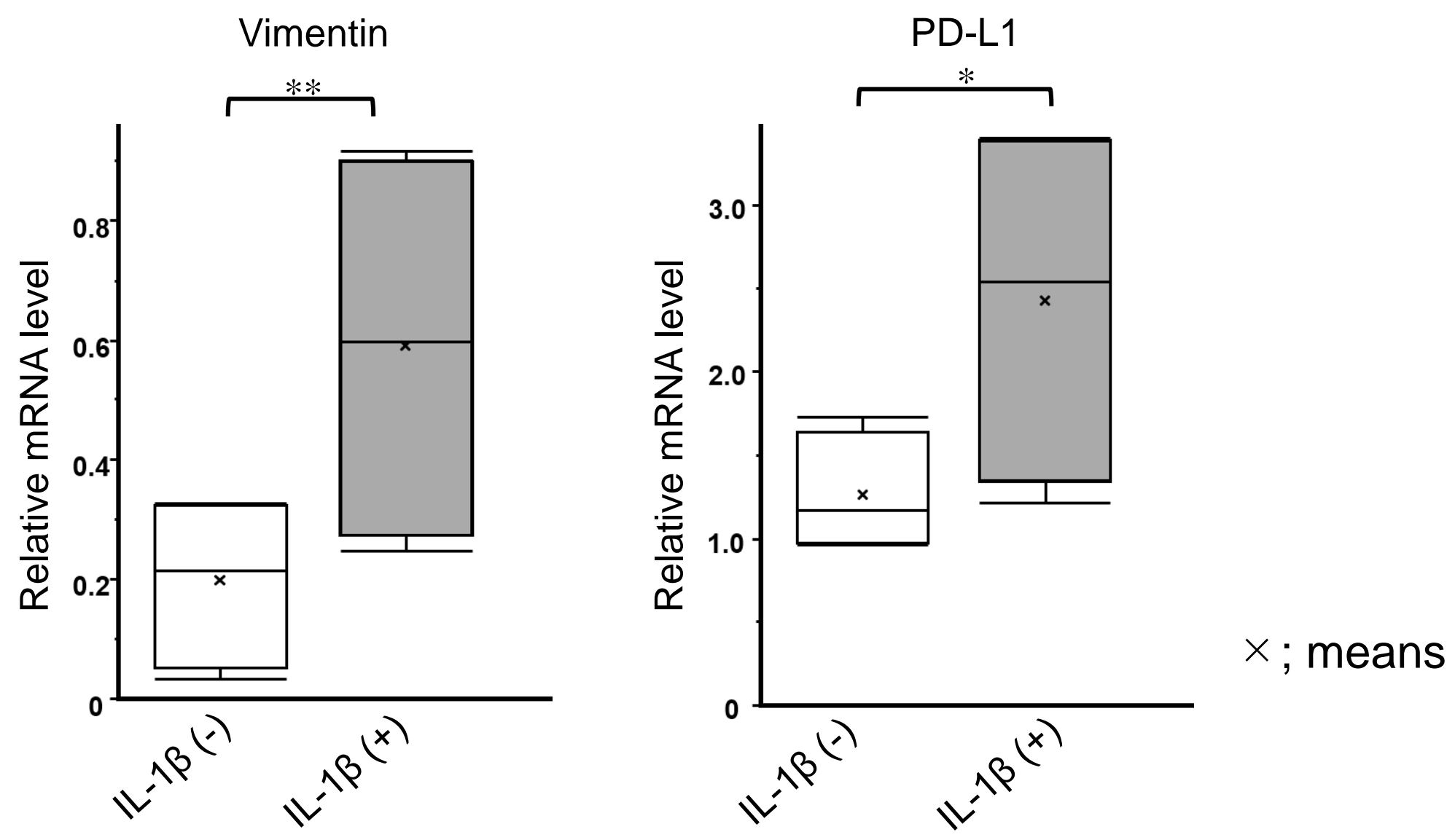

Supplement: Supplementary file 5 — Figure S5. [file JHBP-31-528-s003.pdf]
